# Manual for proposing a Part of the List of Available Names (LAN) in Zoology

**DOI:** 10.3897/zookeys.550.10042

**Published:** 2016-01-07

**Authors:** Miguel A. Alonso-Zarazaga, Philippe Bouchet, Richard L. Pyle, Nikita Kluge, Daphne Gail Fautin

**Affiliations:** 1 Depto. de Biodiversidad y Biología Evolutiva, Museo Nacional de Ciencias Naturales, Jose Gutierrez Abascal 2, E-28006 Madrid, SPAIN; 2 Laboratoire de Biologie des Invertébrés Marins et Malacologie, Muséum National d`Histoire Naturelle, 55, rue Buffon, 75005 Paris, France; 3 Natural Sciences, Bishop Museum, Honolulu, 1525 Bernice Street, 96817 Honolulu, USA; 4 Saint-Petersburg State University, Saint-Petersburg, Russia; 5 Ecology and Evolutionary Biology, and Natural History Museum, University of Kansas, 1200 Sunnyside Avenue, 66045 Lawrence, Kansas, USA

## 1. Introduction

Article 79 of the Fourth Edition of the International Code of Zoological Nomenclature (henceforth Code) describes an official List of Available Names in Zoology (henceforth LAN), consisting of a series of “Parts” (of defined taxonomic and temporal scope), compiled by relevant experts. The LAN represents a comprehensive inventory of names available under the Code. The aim of this manual is to define a procedure for implementing Article 79, with format suggestions for zoologists aiming to create a Part of the LAN for family-group, genus-group, or species-group names in zoological nomenclature. Because the LAN may serve as an important basis for retrospective content in ZooBank, the structure outlined here is designed to allow easy importation to ZooBank.

A Part ultimately adopted for the LAN will contain nomenclaturally available names but not necessarily all those within the scope of the Part: the comprehensiveness of the candidate Part is at the discretion of the experts proposing the Part. They may choose to include all nomenclaturally available names or use the proposal of a Part to pare away *nomina dubia* so they lose “status in zoological nomenclature despite any previous availability” (to quote Articles 10.7 and 79.4.3; that this was the intention of the framers of Article 79 is clear from the Preface to the Code). Nonetheless, we advocate that the proposing body include an inventory of all known names deemed to be available so it will be obvious that names not advocated for inclusion in the Part have not simply been overlooked. Because a candidate Part of the LAN is for an entire taxon at the specified rank and for the specified period, it must include the names of both living and fossil representatives of the taxon.

In the proposal for adding a Part to the LAN, an unavailable name corresponding to a later available one should be included in the Remarks section of the available name. Unavailable names that have not subsequently been made available can be added at the end of the candidate Part, along with information explaining them. The Commission and reviewers of the candidate Part will thereby have a list of such names and an understanding of why they are not available. Moreover, these names can be discussed during the periods required by Article 79 for input by the zoological community, when change in their status can be advocated by members of the community interested in the taxon under consideration.

## 2. Code authority

The consideration of candidate Parts of the LAN is specified by some articles of the Code as well as the Bylaws and Constitution of the International Commission on Zoological Nomenclature (henceforth the Commission). Parallel with this are provisions for the treatment of the Official Lists and Indexes. The relevant portions are reproduced below.

### 2.1. List of Available Names, Official Lists and Indexes

**2.1.1. In the Preface of the Code**

“We can anticipate that zoologists and other users of scientific names will before long require still further changes in the Code, perhaps especially concerning procedures for the listing of existing names and the registration of new ones. With regard to the former, extensive databases are now appearing in quick succession and are being consolidated by such enterprises as Species 2000, and this fourth edition of the Code has already taken a significant step through the provisions for the development and adoption of List(s) of Available Names in Zoology.”

“Progress is made in this edition to establish a mechanism to facilitate access to previously established names, and to achieve certainty that searches made for names are complete, by enabling international groups of specialists to compile lists of extant and known available names in major taxonomic fields, and to have these lists adopted by the Commission. Names not in a relevant adopted List would not be available. A similar policy has already been adopted for all genera and species in microbiology, where neither past nor new names are available unless they have been officially recorded.”

“Lists of Available Names

15. The Commission is empowered, with safeguards, to adopt lists of names in major taxonomic fields. Names within the scope of such an adopted list but not listed in it will be treated as unavailable. Lists may only be adopted by the Commission which have been proposed by international bodies, and only after publication of the proposals, wide consultation with specialist committees and others, and taking into account public comment.”

**2.1.2. In Articles of the Code**

10.7. Availability of names not listed in a relevant adopted Part of the List of Available Names in Zoology. No unlisted name within the scope of an adopted Part of the List of Available Names in Zoology is available, despite any previous availability [Art. 79.4.3].

57.2. Primary homonyms. Identical species-group names established for different nominal taxa when originally combined with the same generic name (see also Articles 11.9.3.2 and 57.8.1) are primary homonyms [Art. 53.3] and the junior name is permanently invalid (but see Article 23.9.5) except when:

57.2.1. its use as a valid name (a nomen protectum) is maintained under the conditions specified in Article 23.9, or

57.2.2. it is conserved by the Commission under Article 81, or

57.2.3. it, but not its senior homonym, is included in a relevant adopted Part of the List of Available Names in Zoology (see Article 79.4.3).

78.2.1. The Commission may, under procedures specified in Article 79, establish a List of Available Names in Zoology and may adopt Parts of the List (for the status of names in the List of Available Names in Zoology, and the name-bearing types of the nominal taxa the names denote, see Article 79.4).

78.4. Other duties. The Commission shall

78.4.2. enter in the relevant Official Lists and Indexes the names and works that have been the subject of rulings by the Commission in its Opinions (including Official Corrections);

Article 79. List of Available Names in Zoology. An international body of zoologists (such as an International Congress, an international society, or a consortium of national or regional societies, or a Scientific Member of the International Union of Biological Sciences) in consultation with the Commission may propose that the Commission adopt for a major taxonomic field (or related fields) a Part of the List of Available Names in Zoology. The Commission will consider the proposal and may adopt the Part subject to the proposing body and the Commission meeting the requirements of this Article.

79.1. Form of the proposal. The proposal to the Commission shall be made in the form of the Part proposed for adoption and shall

79.1.1. specify the scope of the proposal, such as the taxonomic field, ranks, and time period covered, (e.g. Amphibia, Names of the Species Group established before 31 December 1995 [full date, i.e. day, month, year]);

79.1.2. for each name to be listed, give the bibliographic reference to the work in which it is established, its authorship, its date of publication and its status (including its precedence if this is different from its priority);

79.1.3. for each name to be listed, give details of the name-bearing type of the nominal taxon it denotes; in the case of a species-group name, if the details of how the type specimen(s) may be recognized are not known, state whether the name is based on a holotype, syntypes, lectotype or neotype and the place(s) of deposition (if any) recorded in the type fixation (but no lectotype or neotype designation can be made for the purposes of listing alone [Arts. 74.7, 75.3]);

79.1.4. for any name to be listed which has been the subject of a Commission ruling [Arts. 80, 81], give the relevant Opinion and the status of the name as ruled therein; and

79.1.5. if applicable, specify how homonymy with names beyond the scope of the proposal has been resolved.

79.2. Requirements concerning notification, consultation and voting by the Commission.

79.2.1. Upon being advised by an international body of zoologists that it intends to propose a Part of the List, the Commission shall appoint by its Council an *ad hoc* committee [Constitution Art. 10] to consult with the proposers.

79.2.2. Upon receipt of a proposal the Commission shall

79.2.2.1. publish a notice of the proposal in the Bulletin of Zoological Nomenclature giving details of the proposing body, proposed scope of the Part and a source from which copies (on paper or otherwise) of the proposed Part may be obtained by zoologists, and inviting comments from zoologists during the following twelve months;

79.2.2.2. submit the notice for publication in journals publishing taxonomic work in the taxonomic field covered by the proposal;

79.2.2.3. refer the proposal to its *ad hoc* committee for it to receive comments, consult with the proposers and others and, not less than two years from the date of publication of the notice referred to in Article 79.2.2.1, consider either a revised proposal or a recommendation that the proposal be abandoned;

79.2.2.4. ensure that the revised proposal does not contain any name established less than five years before the submission of the initial proposal;

79.2.2.5. following receipt of the revised proposal from its *ad hoc* committee, publish notice of it and invite comments on the revised proposal in the same manner as for the initial proposal [Arts. 79.2.2.1, 79.2.2.2];

79.2.2.6. take into account comments received (if any) and comments of the proposers thereon, and vote to adopt the Part proposed or to abandon the proposal, under procedures prescribed in the Constitution [Art. 12] and the Bylaws of the Commission for voting under its plenary power.

79.3. Effective date of Parts and their accessibility. The Commission shall publish a notice in the Bulletin of Zoological Nomenclature of a decision to adopt any Part of the List of Available Names in Zoology as soon as possible after the decision is taken.

79.3.1. Before publishing the notice of adoption, the Commission shall satisfy itself that the Part newly adopted is accessible either by purchase or gratis and shall include that information in the notice.

79.3.2. Any Part of the List of Available Names in Zoology adopted by the Commission becomes effective from the date of publication in the Bulletin of Zoological Nomenclature of a notice of the decision of the Commission to adopt it.

79.3.3. The notice shall specify the title under which the Part of the List adopted by the Commission shall be known and its scope (including the taxonomic field and dates covered).

79.4. Status of names, spellings, dates of availability, and types specified in the List of Available Names in Zoology.

79.4.1. A name occurring in an adopted Part of the List of Available Names in Zoology is deemed be an available name and to have the spelling, date, and authorship recorded in the List (despite any evidence to the contrary).

79.4.2. A nominal taxon denoted by a name occurring in an adopted Part of the List of Available Names in Zoology is deemed to have the name-bearing type recorded therein (despite any evidence to the contrary).

79.4.3. No unlisted name within the scope (taxonomic field, ranks, and time period covered) of an adopted Part of the List of Available Names in Zoology has any status in zoological nomenclature despite any previous availability.

*Recommendation* 79A. Citation of previously available names. If for taxonomic and historical purposes an author desires to cite a name that is no longer available because it is not included in the relevant Part of the List of Available Names in Zoology adopted by the Commission, it should be made clear that it no longer has a status in zoological nomenclature.

79.5. Power of the Commission to amend the status of a name occurring in the List of Available Names in Zoology. If there are exceptional circumstances and only when an entry in the List of Available Names in Zoology is a cause of confusion, the Commission may amend the entry by use of its plenary power [Art. 81] and publish its ruling in an Opinion [Art. 80.2].

79.5.1. From the date of the publication in the Bulletin of Zoological Nomenclature of the amended entry the relevant name has the status, spelling, date of availability, and authorship, and the nominal taxon it denotes has the name-bearing type, as shown in the amended entry.

79.5.2. The requirement that amendments to the status of names occurring in the List may be made only by the Commission using its plenary power does not prevent an author from designating a type species for a nominal genus-group taxon published before 1931, if one has not already been fixed, or from designating a lectotype [Art. 74] from syntypes recorded in the List of Available Names in Zoology, or a neotype when circumstances exist that require neotype designation [Art. 75]. Such subsequent fixations may be inserted by the Commission in the List.

*Recommendation* 79B. Request to authors designating lectotypes or neotypes for names in the List of Available Names in Zoology. Authors are requested to inform the Commission of lectotype or neotype designations made by them for the nominal taxa of names in the List of Available Names in Zoology as soon as possible after publication.

79.6. Power of the Commission to add omitted names to the List of Available Names in Zoology. If the Commission determines that there is a previously available name within the scope of an adopted Part of the List of Available Names in Zoology that has been omitted from the List, in exceptional circumstances the Commission may by use of the plenary power add an appropriate entry to that Part of the List and record this in an Opinion. The availability of the name thereby becomes restored.

Article 80. Status of actions of the Commission. As a consequence of actions required of it by the Code, the Commission may publish Declarations, Opinions, the Official Lists and Official Indexes, and may adopt and publish Parts of the List of Available Names in Zoology. The status of these published acts, and of names and works in the Official Lists and Official Indexes, is specified in this Article.

80.4. Corrections of errors or omissions in Opinions. Official Corrections to errors and omissions (such as a bibliographic error, lapsus calami, or an omission in placing a conserved or suppressed name on an Official List or Index) may be published by the Commission without further vote unless the error or omission negates the ruling or its consequences. If the ruling is negated by the error or omission, the Commission shall reconsider the matter and publish a further Opinion.

80.6. Status of works, names and nomenclatural acts in Official Lists. The Commission publishes the effects of its Opinions on individual names and works in the Official Lists and Official Indexes. In the case of names and works in the Official Lists:

80.6.1. A name entered in an Official List is an available name.

80.6.2. The status of a name entered in an Official List is subject to the ruling(s) in any relevant Opinion(s), including any Official Correction of an Opinion [Art. 80.4]; all other aspects of its status derive from the normal application of the Code. However, if such a name is given a different status in the List of Available Names in Zoology the latter status is deemed to be correct [Art. 80.8].

80.6.3. A name may be placed in an Official List without any additional qualification.

80.6.4. If a name entered in an Official List is thought to be a synonym of another available name (whether in an Official List or not), their relative precedence is determined by the normal application of the Code unless the Commission rules or has ruled otherwise.

80.6.5. A name or nomenclatural act occurring in a work entered in the Official List of Works Approved as Available for Zoological Nomenclature is subject to the provisions of the Code and to any limitation imposed by the Commission on the use of that work in zoological nomenclature.

80.7. Status of works, names and nomenclatural acts in Official Indexes. The Commission publishes the effects of its Opinions on individual names and works in the Official Lists and Official Indexes. In the case of names and works in the Official Indexes:

80.7.1. A work, name or nomenclatural act entered in an Official Index has the status attributed to it in the relevant ruling(s).

80.7.2. A name or nomenclatural act occurring in a work entered in the Official Index has no availability or validity in zoological nomenclature, unless the Commission by use of its plenary power rules otherwise. However, such a work may be used as a source of information relevant to zoological nomenclature unless the Commission has ruled that the work is to be treated as unpublished.

80.8. Contradictory status accorded by the Commission to names in the List of Available Names in Zoology and in the Official Lists. In the event of contradictory status being accorded by the Commission to a name included in the List of Available Names in Zoology, in an Official List, or in an Opinion, the status accorded in the List of Available Names in Zoology is deemed to be correct unless the Commission has ruled otherwise [Art. 79.5].

**2.1.3. In the Glossary**

*adoption*, n. Of a Part of the List of Available Names in Zoology: the acceptance of the Part by the Commission as specified in Article 79.

*Index, Official*. See Official Index.

*List of Available Names in Zoology*, n. The cumulative term for those parts of the List of Available Names in Zoology which have been adopted by the Commission under Article 79.

*List, Official*. See Official List.

*Official Index*, n. An abbreviated title for any of the four Indexes, maintained and published by the Commission, citing works or names that have been rejected by rulings of the Commission. For the status of names cited in the Indexes, and of names and nomenclatural acts in works cited in the Indexes, see Article 80.7. The full titles of the Indexes are:

*Official Index of Rejected and Invalid Works in Zoological Nomenclature.*

*Official Index of Rejected and Invalid Family-Group Names in Zoology.*

*Official Index of Rejected and Invalid Generic Names in Zoology.*

*Official Index of Rejected and Invalid Specific Names in Zoology.*

*Official List*, n. An abbreviated title for any of the four Lists, maintained and published by the Commission, citing available works or names that have been ruled upon in the Opinions of the Commission. For the status of works, names, and nomenclatural acts in the Lists see Article 80.6. The full titles of the Lists are:

*Official List of Works Approved as Available for Zoological Nomenclature.*

*Official List of Family-Group Names in Zoology.*

*Official List of Generic Names in Zoology.*

*Official List of Specific Names in Zoology.*

(See also List of Available Names in Zoology).

*Part of the List of Available Names in Zoology*, n. (q.v.). A list, adopted by the Commission under Article 79, of available names in a major taxonomic field.

*proposal*, n. (1) An action, whether successful or unsuccessful, to establish a nominal taxon or name or to carry out a nomenclatural act (q.v.). (2) An application to the Commission under Article 79 for the adoption of a Part of the List of Available Names in Zoology.

*rejected work*. Any work included by the Commission in the Official Index of Rejected and Invalid Works in Zoological Nomenclature.

### 2.2. Official Lists in the Bylaws

Mention in the Bylaws, which is under the heading “The Secretariat”, states:

23. The duties of the Secretariat are:

(b) To prepare and edit for publication the Bulletin of Zoological Nomenclature, successive instalments of the official lists and indexes (Constitution Art. 14c), and editions of the Code, Constitution and Bylaws.

### 2.3. Official Lists in the Constitution

Mentions in the Constitution, which are under the heading of Art. 14 “Editorial duties of the Commission”, state:

14.3. Maintenance of Official Lists and Indexes. The Commission shall compile and maintain the under mentioned Lists and Indexes:

*14.3.1. Official List of Family-Group Names in Zoology*;

*14.3.2. Official List of Generic Names in Zoology*;

*14.3.3. Official List of Specific Names in Zoology*;

*14.3.4. Official Index of Rejected and Invalid Family-Group Names in Zoology*;

*14.3.5. Official Index of Rejected and Invalid Generic Names in Zoology*;

*14.3.6. Official Index of Rejected and Invalid Specific Names in Zoology*;

*14.3.7. Official List of Works Approved as Available for Zoological Nomenclature*;

*14.3.8. Official Index of Rejected and Invalid Works in Zoological Nomenclature*.

14.4. *List of Available Names in Zoology*. The Commission may consider, adopt and publish notices concerning the List of Available Names in Zoology (or Parts thereof) as prescribed in Article 79 of the Code.

## 3. Philosophy

Although it is clear from several Articles of the Code that the LAN and the Official Lists and Indexes are different entities, neither it nor the Bylaws and Constitution are very explicit about the differences and the relationship between them. With the help of the Glossary, we make the following clarifications:

### 3.1. LAN

An inventory of names in the Family-, Genus- and Species-groups formed by accumulating Parts as stipulated under Article 79 of the Code and adopted by the Commission. The Code does not provide for an equivalent List for Works.

### 3.2. Official Lists

These four Lists are compiled from the Opinions of the Commission and its use of the Plenary Power. One is related to Works (it is the only way to make available works that otherwise could be doubtfully considered as available). Three others are related to Names and Nomenclatural Acts (one each for Species, Genera, and Families) whose status is ruled by Article 80.6. Their counterparts are the *Official Indexes*, which cite works or names that have been rejected by rulings of the Commission.

### 3.3. Duties of the Commission

The Commission:

- shall compile and maintain the *Official Lists* (and *Indexes*) (Constitution Art. 14.3); enter the names and works that have been the subject of rulings by the Commission in its Opinions (including Official Corrections) (Code Art. 78.4.2);
- may establish a LAN (Code Art. 78.2.1);
- may adopt Parts of it for a major taxonomic field, when proposed by international bodies of zoologists in consultation with the Commission (Code Art. 79);
- shall appoint by its Council an *ad hoc* committee to consult with the proposers (Code Art. 79.2.1, Constitution Art. 10); refer the proposal to its *ad hoc* committee for it to receive comments, consult with the proposers and others and, not less than two years from the date of publication of the notice, consider either a revised proposal or a recommendation that the proposal be abandoned (Code Art. 79.2.2.3);
- shall consider and publish notices concerning the LAN (or Parts thereof) (Constitution Art. 14.4, Code Art. 79):
- shall invite comments on the revised proposal in the same manner as for the initial proposal (Code Arts. 79.2.2.1, 79.2.2.2, 79.2.2.5);
- shall take into account comments received (if any) and comments of the proposers thereon, and vote to adopt the Part proposed or to abandon the proposal, under procedures prescribed in the Constitution [Art. 12] and the Bylaws of the Commission for voting under its plenary power.

### 3.4. Status of names in the LAN

The status of the names in the LAN or its Parts are covered by Article 79.4 of the Code. Names can be amended in (Article 79.5), added to (Article 79.6), or deleted from the LAN by using the Plenary Power and publishing an Opinion. The status of names in the LAN supersede those in the *Official Lists* (cf. Articles 80.6.2, 80.8). Within the scope of period, rank, and taxon for which a Part has been adopted, names not listed are not available (Articles 10.7, 79.4.3).

## 4. Structure of a proposal

### 4.1. Introduction

A proposal for a Part of the LAN should have a Title and an inventory or two inventories of nomenclaturally available names within the scope of the candidate Part, each accompanied by the information required in Articles 79.1.2 through 79.1.5. Whether the names are in one inventory or two separate ones, two categories of names must be clearly distinguished:

1. names proposed for adoption as Part of the LAN; and,
2. names proposed not to be included in the Part, with reasons for their lack of inclusion addressed.

The *ad hoc* committee formed under Article 79.2.1 will determine that the candidate Part does not overlap with any other Part of the LAN (accepted or under consideration) and may propose changes in the taxonomic and temporal limits. During the periods of public discussion (Article 79.2.2.1 and 79.2.2.5), any interested zoologist may comment on the lists, or, by addressing a formal request to the *ad hoc* committee, request modifications, ask that names be transferred between categories, or indicate interest in being consulted further (Article 79.2.2.3).

In the following text, curly brackets { } have been used to include terms that should be replaced by those appropriate to the Part of the LAN being presented.

### 4.2. Title of proposal

Any proposal should be entitled and formatted as follows, as required by Article 79.9.1:

1. **Candidate Part of the List of Available Names in Zoology**
2. **Major taxonomic field**: The name of the taxon, followed by author and date; if needed, the including taxa.
3. **Rank-group**: {Family-, Genus-, Species-} Group Names.
4. **Time period covered**: Before {day month year}. No name established less than 5 years prior to the date of the proposal presentation can be considered (Article 79.2.2.4).
5. **Prepared by**: Authors of the list must provide names, postal addresses, and e-mail addresses.
6. **Presented by**: The international body under the aegis of which this candidate Part of the List was prepared.
7. **Adopted**: Date in which the international bodies presenting the proposal endorsed it (and optionally, place, if in a meeting or congress).
8. **Summary**: An overview of the taxonomic field covered, and possible alternative interpretations if differing taxonomic systems exist (including an overview of related taxa that might be excluded or included in alternative systems). This summary must include the taxonomic criteria followed in compiling the list, citing references or sufficient details to be clear, if the criteria are new or differ considerably from those published. The number of taxa proposed for adoption as Part of the LAN, and the number proposed not to be in the Part (as per § 4.1 above).

### 4.3. Family-group names

For the purpose of compiling a Part of the LAN, a family-group name is any uninominal, plural noun or nominalized adjective (Arts. 4.1, 11.7.1, 29). It may be formed by adding to the stem of a valid type genus name any suffix and/or ending (Arts. 11.7, 29, 63, 64), even if not standard under the Code (Arts. 11.7, 29); originally it can be attributed to any rank included in the family- group in the system followed by its author or have an unnamed rank within it (Art. 35). In publications issued when the family group did not exist (e.g., prior to 1802) or in those where family group names are not accepted, any rank above genus group may be regarded as belonging to the family group. Names clearly attributed to a family-group rank but not based on a type genus name must also be considered, although only for rejection.

The fields of a candidate Part of the LAN in the Family-Group are:

**Stem**: The proposed stem to be followed by standard (and non-standard) endings, according to Article 29 of the Code. The stem must be written in small caps, and capitalized.
**Original spelling**: The spelling as originally written, including diacritics, if present. Use small caps, and capitalize. If in the same work a name appears in Latinized and vernacular forms, the Latinized form is preferred. If the name appears in different ranks, the highest rank within the family-group has precedence (Article 24.1) as the origin; others must be mentioned in the Remarks field. Care should be taken not to confuse faulty (or non-standard) Latin with vernacular spelling. Consider also Articles 32.5.3.2 and 35.4.1 for names formed from incorrect subsequent spellings or unjustified emendations (but see Article 35.4.2).
**Author**: The name(s) of the author(s) of the taxon.
**Year**: The year of effective publication.
**Reference**: In the format of the Bulletin of Zoological Nomenclature and the Official Lists and Indexes of Names and Works in Zoology, Supplement 1986-2000.
**Rank**: The original explicit rank, if any. If not explicit or the rank is not a standard one (family, tribe; with prefixes: super-, sub-, infra-; prefixes may be combined), state: “Unnamed rank intended to be between {rank_1} and {rank_2}” or “Unnamed rank intended to be immediately above or below {rank}”. Non-standard rank names can be used instead of “Unnamed rank”. If a name given to a phylogenetic clade is also available for nomenclatural purposes under the present Code, it is to be stated as: “Clade child of {clade name_1} and sister to {clade name_2}”.
**Type genus**: The valid name of the type genus in **bold font**. If the original spelling was based on an unjustified emendation or on an incorrect subsequent spelling, that must be given in the Remarks field.
**Type genus author**: The name(s) of the author(s) of the genus name.
**Type genus year**: The year of effective publication of the genus name.
**Type genus reference**: In consistent format (see 5 above).
11. **Type genus rank in original description**: Originally described as genus or subgenus of {Genus Author, Year}.
12. **Latinization**: Reference to first latinization for an originally vernacular name, quoting the first author to latinize it and the reference. If original, state “Original”. If not so, following Art. 11.7.2, it should be added: “Generally accepted as valid by authors interested in the group concerned and as dating from that first publication in vernacular form”. Note that first latinization has not to be standard, according to the present Code.
13. **Qualification**: Include statements about availability and precedence. First Revisers’ actions regarding the precedence of names published at the same time must be quoted here, including data on precedence if different from priority (Article 79.1.2). Include information on how homonymy with names beyond the scope of the proposal has been resolved, if applicable (Article 79.1.5). If relevant, statements about the application of Articles 13.2.1 and 40 to the name are expected.
14. **Previous rulings**: Quote in full any ruling (Opinion or Direction) by the Commission on the name and how it is affected by them, including placement on Official Lists or Indexes (Article 79.1.4).
15. **Remarks**: Add any comment to clarify the status of the name or other relevant data.

### 4.4. Genus-group names

For the purpose of compiling a candidate Part of the LAN, a genus-group name is any uninominal, singular noun or nominalized adjective, regarded to be Latin or latinized, corrected to nominative singular if given in any case other than this because of the requirements of Latin grammar in Latin texts, originally proposed either for a taxon at the rank of genus or subgenus (Article 42.1), or for a taxon attributed to any rank included in the genus-group in the system followed by its author, or having an unnamed rank within it in terms of the Code.

The fields of a candidate Part of the LAN in the Generic Group are:

1. **Name**: Use **bold font**, and capitalize. Do not use diacritics and other marks; incorrect original spellings must be given in the Remarks field.
2. **Author**: The name(s) of the author(s) of the taxon.
3. **Year**: The year of effective publication.
4. **Reference**: In the format of the Bulletin of Zoological Nomenclature and the Official Lists and Indexes of Names and Works in Zoology, Supplement 1986-2000.
5. **Gender**: Masculine, feminine, or neuter.
6. **Kind of type species designation**: By original designation, by monotypy, by absolute tautonymy, by Linnaean tautonymy, by subsequent designation, by subsequent monotypy, or under the plenary power.
7. **Reference if subsequent**: If the type species designation was subsequent or made under the plenary power, the data for this designation must be provided in consistent format.
8. **Rank**: The original explicit rank, if any. If not explicit or the rank is not standard (genus, subgenus), a statement should be included such as: “Unnamed rank intended to be between {rank_1} and {rank_2}” or “Unnamed rank intended to be immediately above/below {rank}”. Non-standard rank names can be given instead of “Unnamed rank”. If a name given to a phylogenetic clade is also available for nomenclatural purposes, it is to be stated as: “Clade child of {clade name_1} and sister to {clade name_2}”.
9. **Type species**: The name of the type species, in its original combination, using **bold font**. If the original spelling was an unjustified emendation or an incorrect subsequent spelling, that must be given in the Remarks field.
10. **Type species author**: The name(s) of the author(s) of the species name.
11. **Type species year**: The year of effective publication of the species name.
12. **Type species reference**: In consistent format (see 4 above).
13. **Type species proposed valid name**: The proposed senior synonym of the type species under the taxonomic system used, in its valid combination [Genus (Subgenus) species Author, Year], if the type species is currently considered to be a junior synonym, using **bold font**.
14. **Qualification**: Include statements about availability and precedence. First Revisers’ actions regarding the precedence of names published at the same time must be quoted here, including data on precedence if different from priority (Article 79.1.2). Statements about stems for the formation of family-group names are to be placed here. Include information on how homonymy with names beyond the scope of the proposal has been resolved, if applicable (Article 79.1.5).
15. **Previous rulings**: Quote in full any ruling (Opinion or Direction) by the Commission on the name and how it is affected by them, including placement on Official Lists or Indexes (Article 79.1.4).
16. **Remarks**: Add any comment to clarify the status of the name or other relevant data.

### 4.5. Species-group names

For the purpose of compiling a Part of the LAN, an available species-group name should be a specific name, regarded to be a Latin or latinized adjective, participle or noun in nominative singular or genitive case (Art. 11.9.1), satisfying the provisions of the Code; it must be originally published in unambiguous combination with a generic name (Art. 11.9.3) and originally proposed either for a taxon at the rank of species or subspecies (Art. 45.1), or published before 1961 for a “variety” or “form” (Art. 10.2) not deemed to be infrasubspecific (Art. 45.6).

The fields of a candidate Part of the LAN in the Specific Group are:

1. **Specific name**: The specific epithet as it should have been correct in the original publication, if any correction of the original spelling is mandatory (Articles 32.5, 33.2.2), using **bold font**. Incorrect original spellings must be given in the Remarks field.
2. **Genus name**: The original genus name as originally spelled, with corrected spelling if originally misspelled, using **bold font**. Incorrect original spellings must be given in the Remarks field.
3. **Author**: The name(s) of the author(s) of the taxon.
4. **Year**: The year of effective publication.
5. **Reference**: In the format of the Bulletin of Zoological Nomenclature and the Official Lists and Indexes of Names and Works in Zoology, Supplement 1986-2000.
6. **Rank**: The original explicit rank, if any. If not explicit or the rank is not a standard one (species, subspecies), a statement should be included such as: “Unnamed rank intended to be between {rank_1} and {rank_2}” or “Unnamed rank intended to be immediately above/below {rank}”. Non-standard rank names can be given instead of “Unnamed rank”. If a name given to a phylogenetic clade is also available for nomenclatural purposes, it is to be stated as: “Clade child of {clade name_1} and sister to {clade name_2}”.
7. **Type specimen(s) data**: State details of how the type specimen(s) may be recognized, including, if relevant, reference to a definition or interpretation such as “designated by” or “figured by”, in consistent format. If such details are unknown, it should be stated whether the name is based on a holotype, syntypes, lectotype, or neotype, and the place(s) of deposition (if any) recorded in the type fixation (but no lectotype or neotype designation can be made for the purposes of listing alone [Articles 74.7, 75.3]) (Article 79.1.3). If the type specimen(s) ha(s/ve) been the subject of an Opinion or Declaration to define the taxon, state “as defined by the {neotype, lectotype, holotype}… in {depository}” or “as interpreted by reference to the {neotype, lectotype, holotype}”.
8. **Qualification**: Include statements about availability and about validity under the taxonomic system used. First Revisers’ actions regarding the precedence of names published at the same time must be quoted here, including data on precedence if different from priority (Article 79.1.2). State also whether the species is the type species of a genus: “specific name of the type species of {Genus Author, Year}”. Include information on how homonymy with names beyond the scope of the proposal has been resolved, if applicable (Article 79.1.5).
9. **Previous rulings**: Quote in full any ruling (Opinion or Direction) by the Commission on the name and how it is affected by them, including placement on Official Lists or Indexes (Article 79.1.4).
10. **Remarks**: Add any comment to clarify the status of the name or other relevant data.

## 5. References

The citations follow the standard in the Bulletin of Zoological Nomenclature.

Authors must furnish a separate list of the references used, formatted as the bibliographic sections of the Bulletin. Each reference should have a day-month-year field (as far as discernable) for publication date to help with priority issues.

